# Influenza A Virus Induces an Immediate Cytotoxic Activity in All Major Subsets of Peripheral Blood Mononuclear Cells

**DOI:** 10.1371/journal.pone.0004122

**Published:** 2009-01-06

**Authors:** Sanda Sturlan, Monika Sachet, Suzann Baumann, Irina Kuznetsova, Andreas Spittler, Michael Bergmann

**Affiliations:** Division of General Surgery, Department of Surgery, Medical University of Vienna, Vienna, Austria; New York University School of Medicine, United States of America

## Abstract

**Background:**

A replication defective influenza A vaccine virus (delNS1 virus) was developed. Its attenuation is due to potent stimulation of the innate immune system by the virus. Since the innate immune system can also target cancer cells, we reasoned that delNS1 virus induced immune-stimulation should also lead to the induction of innate cytotoxic effects towards cancer cells.

**Methodology/Principal Findings:**

Peripheral blood mononuclear cells (PBMCs), isolated CD56+, CD3+, CD14+ and CD19+ subsets and different combinations of the above subsets were stimulated by delNS1, wild type (wt) virus or heat inactivated virus and co-cultured with tumor cell lines in the presence or absence of antibodies against the interferon system. Stimulation of PBMCs by the delNS1 virus effectively induced cytotoxicity against different cancer cell lines. Surprisingly, virus induced cytotoxicity was exerted by all major subtypes of PBMCs including CD56+, CD3+, CD14+ and CD19+ cells. Virus induced cytotoxicity in CD3+, CD14+ and CD19+ cells was dependent on virus replication, whereas virus induced cytotoxicity in CD56+ cells was only dependent on the binding of the virus. Virus induced cytotoxicity of isolated cell cultures of CD14+, CD19+ or CD56+ cells could be partially blocked by antibodies against type I and type II (IFN) interferon. In contrast, virus induced cytotoxicity in the complete PBMC preparation could not be inhibited by blocking type I or type II IFN, indicating a redundant system of activation in whole blood.

**Conclusions/Significance:**

Our data suggest that apart from their well known specialized functions all main subsets of peripheral blood cells also initially exert a cytotoxic effect upon virus stimulation. This closely links the innate immune system to the adaptive immune response and renders delNS1 virus a potential therapeutic tool for viro-immunotherapy of cancer.

## Introduction

Influenza A virus effectively induces the adaptive and the innate immune response. Stimulation of the adaptive immune response has been regarded as substantial for viral clearance for a long time. In contrast, the relevance of the stimulation of the innate immune system for viral clearance became recently evident when Gazit et al. demonstrated that mice lacking the natural killer (NK) cell receptor Ncr-1 die from otherwise non-lethal influenza A virus infection [1]. NK-cells which are stimulated by the viral hemagglutinin are regarded as the main protagonists of virus induced cytotoxicity of the innate immune system. However, innate cytotoxicity does not seem to be restricted to NK-cells. A plasmacytoid dendritic cell line is also able to exert a cytotoxic effect after contact with influenza A virus [2]. Thus, different cell types might contribute to virus induced innate cytotoxicity.

Apart from the relevance for viral infections, the innate immune system also provides multiple ways of tumor cell destruction due to its potency of effective cytotoxicity towards degenerated cells. It was possible to treat murine cancer by adoptive transfer of splenocytes, bone marrow cells or enriched peripheral macrophages from cancer resistant CR mice [3]. The tumor ablative effect of leukocytes is not restricted to a single cell type but transfer of multiple subsets of leukocytes [4] can promote cytotoxic effects. Tumor ablation by induction of the innate immune system has already led to clinical success. Stimulating toll like receptor (TLR) 7/8 with imiquimod leads to therapeutic effects on basaliomas. This appears to be mediated by cytotoxic activity of dendritic cells present in the tumor tissue [5]. Thus, efficient stimulation of cells of the innate immune system might offer a therapeutic window for cancer therapy in humans [6].

Therapeutic stimulation of the innate immune system may also be induced by viruses. Specifically, oncolytic viruses-viruses which have been generated to conditionally replicate in the tumor tissue [7]-might exert such effects [8]. We have previously developed the first prototype of an oncolytic influenza A virus, based on a deletion of the viral non-structural NS1 gene. This genotype restricts the virus to replication in protein kinase R (PKR)- and IFN- defective tumor cells but not in normal cells [9], [10]. In general, influenza A viruses might be attractive immune stimulants since they effectively activate the endosomal TLR 3 and 7 [11], [12]. In addition to this property, NS1 deletion viruses have a specific immune stimulatory phenotype, since the virus lacks its natural viral antagonist of the IFN pathway. Consequently, the NS1 deletion virus effectively induces cytokines of the innate immune system such as TNF, IL-1β and type I IFN [13]–[15]. The induction of those cytokines is explained by the lack of inhibition of RIG-I [16]. Due to this immune stimulatory property and its attenuated nature [17] the NS1 deletion virus seems specifically attractive for viro-immunotherapy of cancer patients.

For example, Efferson et al. [18] have shown, that a prostate tumor cell line infected with influenza virus expressing a truncated NS1 protein activates cytolytic CD8+ cells to recognize non-infected tumor cells. Activation of those antigen-specific CD8+ cytotoxic T-lymphocytes by virus infected tumor cells was achieved by 6 days of co-culture. This stimulation of the adaptive arm of the immune system was due to the inherent property of the virus to induce cross-presentation.

Here we tested whether direct stimulation of PBMCs by the virus is also able to induce an immediate innate cytotoxic response to cancer cells. Surprisingly, we found that virus induced innate cytotoxicity is not restricted to NK-cells or monocytes. Immediate cytotoxic activity was also observed in CD3+ T-lymphocytes and in CD19+ B-lymphocytes. The latter effect appears to largely depend on viral replication and was partially attenuated by antibodies against type I and type II interferons. Our results indicate that viral infection stimulates all major subsets of PBMCs to exert an immediate cytotoxic function.

## Results

### PBMCs stimulated by influenza A NS1 deletion virus exert cytotoxic effects on cancer cell lines

**Figure 1:**
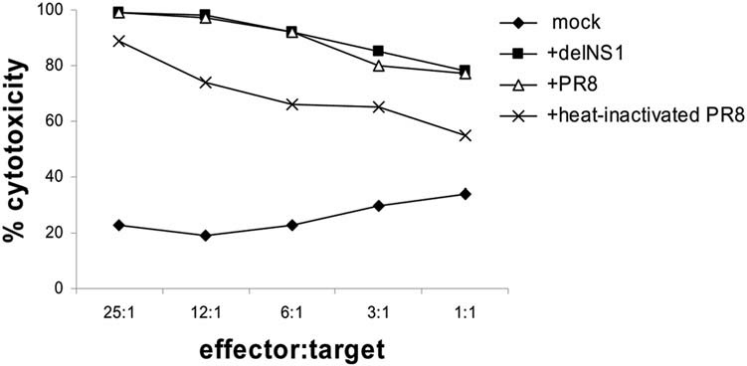
Induction of an anti-tumor cytotoxic immune response by PBMCs infected with influenza A variants. PBMCs were mock-treated or infected with delNS1 virus, wt PR8 and heat-inactivated wt PR8 (m.o.i. = 0.02) and were incubated for 24 hours. Then PBMCs were co-cultured with the target A375 cells for 24 hours and cytotoxicity was assessed thereafter. One representative result out of three independent experiments, each performed with PBMCs derived from a different donor, is shown.

**Figure 2:**
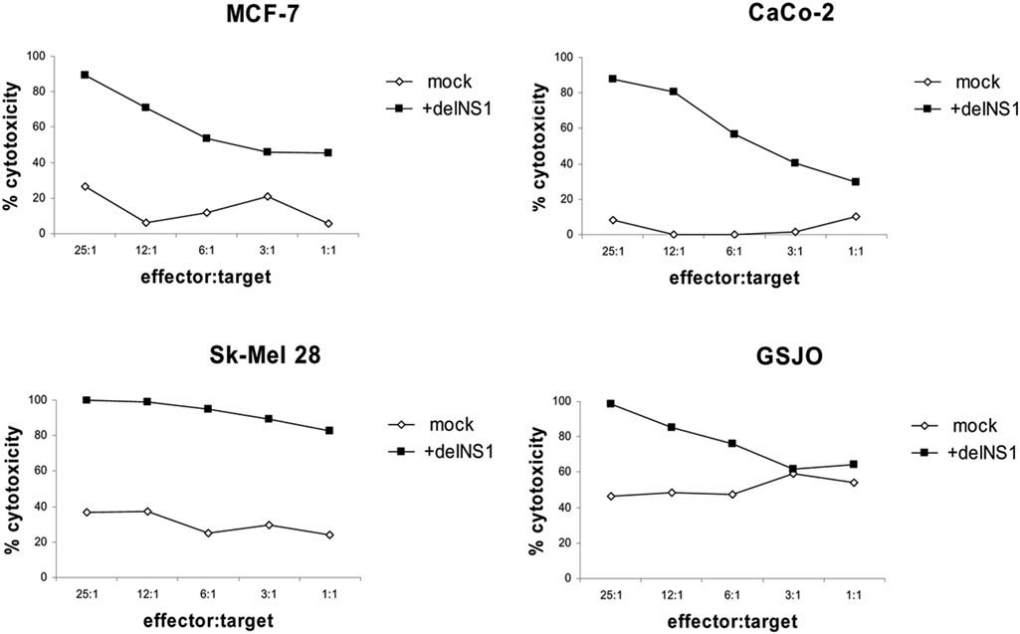
Effect of mock-treated or delNS1 infected PBMCs on tumor cells of different tissue origin. PBMCs were infected with delNS1 virus (m.o.i. = 0.02) and kept for 24 hours. Then the PBMCs were co-cultured with the target cells MCF-7, CaCo-2, Sk-Mel 28 or GSJO for 24 hours and cytotoxicity was assessed thereafter. One representative result in percentage of cytotoxicity is shown out of two independent experiments, each performed with PBMCs derived from a different donor.

First we investigated whether PBMCs of healthy donors can be stimulated by ex vivo infection with wild-type virus versus replication defective influenza A delNS1 virus to induce anti-tumor cytotoxicity. Since multiple publications have shown that cell-cell interactions between immunologically active cells are important for efficient immune-stimulation, it seemed vital to let these cell-cell interactions occur in a total PBMC population. PBMCs were infected/stimulated with wt PR8 influenza virus, heat-inactivated wt PR8 influenza virus or delNS1 virus at a low multiplicity of infection (m.o.i.) of 0.02. As shown in fig. 1 virus-stimulated PBMCs generally displayed a strong cytotoxic effect against the melanoma cell line A375 as compared with non-infected PBMCs. This effect was reproducible for PBMCs isolated from different donors. Importantly, there was no difference in the stimulatory capacity between wt influenza virus and replication defective delNS1 virus, whereas the heat-inactivated wt virus was 10–20% less effective. We then tested whether delNS1 stimulated PBMCs are also able to kill other tumor cell lines such as MCF-7, a human breast cancer cell line, CaCo-2, a human colon cancer line, SK-Mel28, another human melanoma line and GSJO, a human medullary thyroid carcinoma cell line. All targets were efficiently killed by virus activated PBMCs (fig. 2).

**Figure 3:**
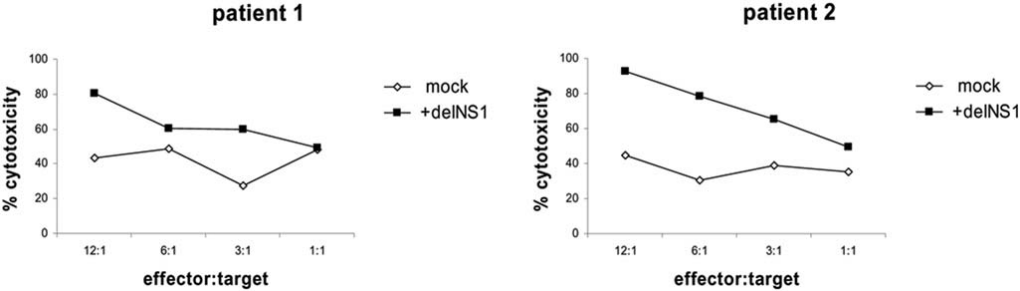
Induction of an anti-tumor cytotoxic immune response by PBMCs derived from patients suffering from medullary thyroid carcinoma. PBMCs were infected with delNS1 virus (m.o.i. = 0.02) or mock-treated and incubated for 24 hours. Then the PBMCs were co-cultured with the target A375 cells for 24 hours and cytotoxicity was assessed. Results in percentage of cytotoxicity are shown for two independent experiments.

Since cancer is an immune-compromised status, we wondered whether infection of PBMCs from cancer patients would also stimulate the immune response against tumor cells. For this purpose we isolated PBMCs from 2 patients suffering from medullary thyroid carcinoma, stimulated these cells with delNS1 as described above and co-cultured them with the primary medullary thyroid carcinoma cell line GSJO. Infection of PBMCs with delNS1 in both patients increased cytotoxic effects against tumor cells, in comparison to non-infected control PBMCs (fig. 3).

**Figure 4:**
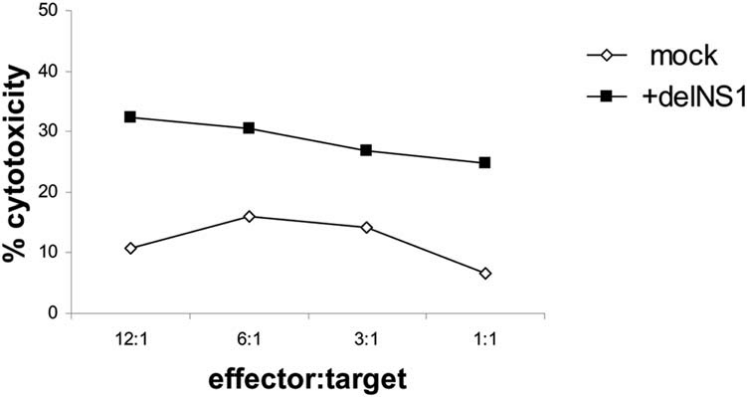
Induction of an anti-tumor cytotoxic immune response by virus infected splenocytes from BALB/c mice against a syngeneic tumor. Splenocytes were mock-treated or infected with delNS1 virus (m.o.i. = 0.02) and incubated for 24 hours. Then splenocytes were co-cultured with the target C-26 cells for 24 hours and cytotoxicity was assessed thereafter. One representative result in percentage of cytotoxicity is shown from three independent experiments.

All of the above described experiments with human PBMCs were performed in the allogeneic system. In this setting virus-enhanced cytotoxic activity of PBMCs against tumor cells could be primarily based on the HLA-mismatch. To evaluate this possibility we performed cytotoxicity experiments in a mouse autologous tumor system. Purified splenocytes from BALB/c mice were infected with delNS1 (m.o.i. = 0.02) which led to a significant induction of cytotoxicity of spleen cells against syngeneic C-26 colon carcinoma cells as compared to non-activated splenocytes (fig. 4).

### Cellular contact and soluble factors are relevant for delNS1 virus mediated anti-tumor cytotoxicity

**Figure 5:**
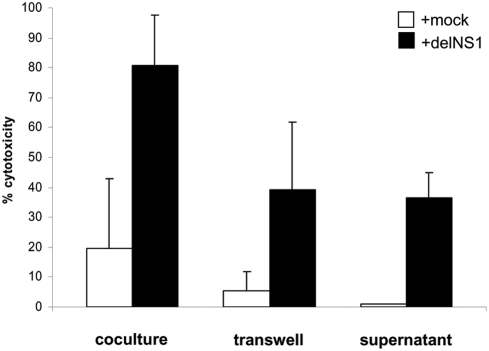
Effect of mock-treated or infected PBMCs on tumor cells under different co-culture conditions. PBMCs were infected with delNS1 virus (m.o.i. = 0.02) or mock-treated and incubated for 24 hours. Then the PBMCs were either co-cultured directly with the target A375 cells (co-culture) or were kept without cellular contact to target cells (transwell). Alternatively, target cells were only supplied with the 24 hour supernatant of PBMC cultures (supernatant). The effector to target ratio was 25:1. After 24 hours cytotoxicity was assessed. Mean results (±SD) in percentage of cytotoxicity are shown from three independent experiments, each performed with PBMCs derived from a different donor.

To analyze whether delNS1 induced cytotoxicity requires cellular contact between target cells and PBMCs, virus-activated PBMCs and A375 melanoma cells were separated by a semi-permeable membrane in a transwell system and co-cultured in the effector to a target ratio of 25:1. The virus-induced cytotoxic effect was reduced to 50% when the cells were separated (fig. 5), demonstrating that the cytotoxic effect of PBMCs depended on both cellular contact and soluble factors. In order to investigate whether those soluble cytotoxic factors are produced upon viral infection and/or whether they require communication (feedback) between tumor-derived soluble factors and PBMCs during the 24 hours of co-culture, we performed an additional experiment. We treated tumor cells solely with the supernatant derived from PBMCs 24 h post infection with delNS1 virus. No difference between the transwell system and the supernatant-based experiment was observed, arguing against the feedback circle (fig. 5).

Since cytotoxicity is partially mediated by PBMC- supernatant we further investigated whether viral progeny could be involved in the cytotoxic effect. We first determined whether infectious viral particles are present in the supernatant of PBMCs infected with delNS1 virus. We found that 5×10^6^ delNS1 infected PBMCs release on average 25 infectious particles into the supernatant. This would correspond to 0.6 infectious particles present in the co-culture experiments done at the 25:1-effector:target ratio, corresponding to an m.o.i.<0.0001. Although this amount of infectious virus particles was very low, we further determined whether delNS1 virus is able to lyse tumor cells in our setting. We used a more than 100-fold higher concentration of the virus and incubated A375, SK-Mel 28, CaCo-2 and MCF-7 tumor cells with delNS1 at the m.o.i. of 0.02 and 0.2. We did not observe any cytotoxicity in the cancer cell lines (fig. S1). To further rule out a possible contribution of progeny virus we performed a co-culture experiment of PBMCs and cancer cells in the presence of a neutralizing anti-influenza antibody. The delNS1-virus stimulated PBMCs induced the same level of cytotoxicity towards tumor cells irrespective of the presence of neutralizing antibody (fig. S2).

### Characterization of immunological markers in delNS1 virus infected PBMCs

**Figure 6:**
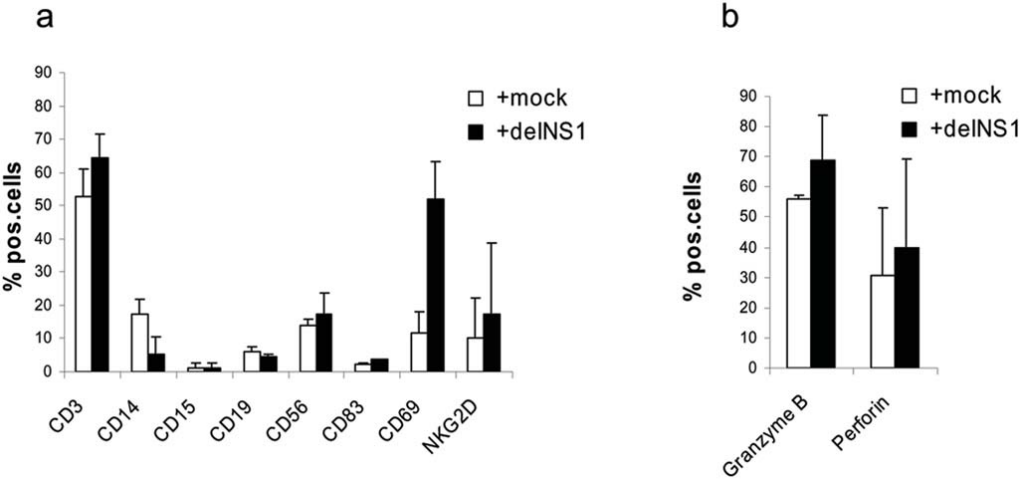
Expression of extracellular and intracellular markers in PBMCs after infection with delNS1 virus. (a) Surface marker expression of mock-treated or infected PBMCs with delNS1 virus was evaluated. PBMCs were infected with delNS1 virus (m.o.i. = 0.02) or not infected and cultured for 24 hours. Then the indicated markers were assessed by flow cytometry using specific fluorescent antibodies. Mean results and standard deviation for the percentage of positive cells are shown from three independent experiments, each performed with cells derived from a different donor. (b) Intracellular marker expression of comparably treated PBMCs was measured after antibody staining of permeabilized cells. Mean results (±SD) for the percentage of positive cells are shown from three independent experiments, each performed with cells derived from a different donor.

We next determined whether delNS1 virus led to an increase of specific subsets of PBMCs. PBMCs were stimulated with the virus and analyzed 24 hours post infection by flow cytometry. As shown in fig. 6a the number of lymphocytes did not change dramatically after infection with delNS1 influenza virus. A significant alteration in response to viral infection was only observed for the CD14+ population, which was more diminished in the virus treated group as compared to the untreated group. No cell population was considerably augmented by the virus. Although the overall number of lymphocyte counts did not change substantially, the expression of CD69 did. This activation marker demonstrated a considerable increase to 52% after viral infection as compared to 12% in non-infected cells. The expression of the NK receptor NKG2D was not significantly increased in response to the virus.

Furthermore, we determined the intracellular expression of the apoptosis inducing molecules granzyme B and perforin (fig. 6b). Although delNS1 virus triggered an up-regulation of these molecules in PBMCs derived from some individuals there was no significant rise of mean levels. The individual up-regulation did not correlate with the intensity of the cytotoxic effect of the respective PBMCs against tumor cells.

The delNS1 virus has been shown to substantially induce pro-inflammatory cytokines such as IL-1β, TNF, IFNα, IL-6, and IFNγ in primary monocytes [14], [19] and/or dendritic cells [15]. We confirmed a similar cytokine pattern in virus induced PBMC cultures. The delNS1 virus induced high levels of IFNα, IL-6, IL-8, IFNγ, TNFα and IL-1β, low levels of IL-10 but no IL-12p70 and no IL-4 (data not shown).

### The role of PBMC subsets in delNS1 virus induced cytotoxicity

In previous reports CD56+ cells have been associated with a strong cytotoxic activity in influenza A virus infection. Moreover, cytotoxic effects of immune-mediators produced by CD14+ cells have been described for other viruses. CD3+ cells are generally known as cytotoxic subsets with regard to adaptive immunity. Therefore we initially focused on these three subsets. In order to evaluate which subsets of cells are important for the delNS1 induced anti-tumor cytotoxicity, we next conducted effector cell depletion experiments. Depletion of CD3+, CD14+ or CD56+ cells was performed before infection with delNS1, using magnetic beads labeled with the respective antibodies. To verify depletion, the remaining cell population was analyzed by flow cytometry which consistently revealed purification grades of >95%. Depletion of a single subset had no effect on delNS1 induced cytotoxicity of PBMCs against tumor cells (data not shown).

We then performed double depletion experiments of selected cell types. Depletion of CD14+ and CD56+ (<1% CD14+ and <2% CD56+) as well as depletion of CD14+ and CD3+ (<3% CD14+ and <15% CD3+) was still associated with killing activity of more than 80% as compared to total PBMCs. Depletion of CD3+ and CD56+ (<2% CD3+ and <1% CD56+) reduced killing to 50% (data not shown).

**Figure 7:**
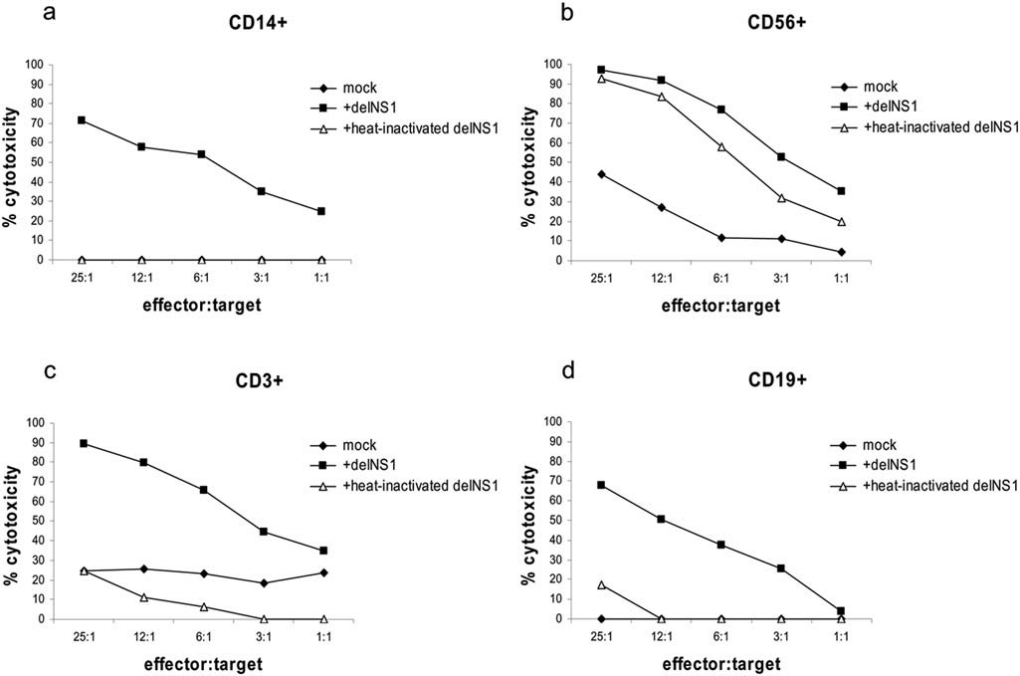
Induction of an anti-tumor cytotoxic immune response by infected monocultures. CD14+ (a), CD56+ (b), CD3+ (c) and CD19+ cells (d) were infected with delNS1 virus or heat-inactivated delNS1 (m.o.i. = 0.02) and incubated for 24 hours. Then the cells were co-cultured with the target A375 cells for 24 hours and cytotoxicity was assessed. One representative result in percentage of cytotoxicity is shown of three independent experiments, each performed with cells derived from a different donor.

According to the data above it seemed likely that more than two of the major cell fractions of PBMCs contribute to delNS1 virus induced cytotoxicity to cancer cell lines. Therefore we determined the cytotoxic effect for each subset separately. For this purpose cells were positively selected by antibody-coupled magnetic beads and afterwards stimulated with the virus. Surprisingly, after 24 hours of co-culture with the melanoma cell line A375, not only CD56+ and CD3+ cells, but also CD19+ and CD14+ cells induced cytotoxicity (fig. 7). Since CD3+ selected cells still contained the CD3+CD56+ NKT-cells, we also included a CD56+ depletion step after CD3+ selection resulting in >90% CD3+ and <3% CD56 positive cells as verified by flow cytometry. The purified T-cell population (CD3+ CD56-) showed the same level of cytotoxicity as the mixture of CD3+CD56- and CD3+CD56+ cells (data not shown). Stimulation of single cell fractions with heat-inactivated virus, which binds to the viral cell receptor but cannot replicate, still induced cytotoxicity in CD56+ cells but not in CD14+, CD19+ or CD3+ cells, indicating that live virus is essential for activation of the latter cell types. CD69 expression was induced by live virus in CD19+, CD56+ and CD3+ cells but not by heat-inactivated delNS1 virus (table 1). Thus, CD69 expression corresponds to live virus infection but not necessarily to virus induced cytotoxicity.

**Table 1:** CD69 expression of monocultures after virus infection.

sorted cells	treatment	CD69 expression (MFI)^a^
**CD56+**	mock	0.9+/−0.2
	delNS1	10.4+/−1.4
	delNS1 inactivated	2.5+/−0.5
**CD3+**	mock	1.5+/−0.5
	delNS1	5.6+/−2.4
	delNS1 inactivated	2.4+/−0.6
**CD19+**	mock	2.3+/−0.9
	delNS1	9.8+/−1.8
	delNS1 inactivated	3.8+/−0.8

a CD69 expression was measured by flow cytometry. Indicated numbers are the mean intensity of fluorescence (MFI) and standard deviation. Two different experiments for each cell type were carried out.

### Analysis of mediators involved in delNS1 virus induced cytotoxicity

Using stimulating virus at low m.o.i. virus stimulated cytotoxicity also depends on a second wave of cytokine activation expressed by non-infected cells. We therefore first determined whether there was a specific uniform cytokine pattern associated with virus induced cell activation of monocultures. We could not detect IL-2, IL-4, IL-5, IL-10, IL-12(p70) and IL-13 above background levels in any of the monocultures. IFNα was produced by all cell fractions except the CD3+ lymphocytes. The latter did not produce any of the here tested common cytokines related to immune activation. It should be noted that plasmacytoid dendritic cells, which are known to generate large quantities of IFNα in the PBMCs, are not present in the here analyzed monoculture fractions, since cells were positively selected by antibodies to CD3, CD14, CD56 or CD19. IFNγ was only produced in high amounts by CD56+ cells, suggesting a possible contribution of this cytokine in activation of this subpopulation. ΤΝF was produced by CD14+ cells and to a lesser extend by CD19+ and CD56+ cells. IL-6 showed a similar expression pattern as IFNα in monocultures (table 2). In PBMCs all cytokines which were also found in monocultures (TNF, IL-6, and type I and type II IFN) were induced to high levels, indicating that cell-cell interaction and dendritic cells present in PBMCs modified the cytokine pattern. A comparison of the absolute amounts of cytokines found in PBMCs with the amounts found in isolated monoculture is difficult, since the number of cells of a defined cell type in PBMCs is less than the number of cells in the monocultures. However, a comparison indicates that high levels of cytokines present in PBMCs are not critically essential for activation of cytotoxicity, since they are not found in monocultures. Moreover, a comparison suggests possible contributions of single subsets to the cytokine response in the complete set of PBMCs.

**Table 2:** Cytokine production after virus infection of CD19+, CD14+, CD56+, CD3+ monocultures and PBMCs.

Cell culture	TNF^a^	IFNγ^a^	IFNα^a^	IL-6^a^
**CD19+**	0	5+/−2	18+/−1	48+/−1
**CD19+delNS1**	105+/−98	5+/−2	134+/−81	161+/−90
**CD14+**	2+/−1	0	19+/−2	51+/−13
**CD14+delNS1**	798+/−254	9+/−2	109+/−16	447+/−164
**CD56+**	7+/−2	9+/−6	17+/−1	16+/−7
**CD56+delNS1**	46+/−27	2622+/−230	150+/−35	104+/−3
**CD3+**	0	5+/−4	18+/−1	8+/−7
**PBMC**	0	18+/−15	106+/−27	28+/−6
**PBMC+delNS1**	340+/−175	4279+/−542	5276+/−225	792+/−492

a Determined by Luminex® and ELISA from supernatants 24h after infection in pg/ml and representing the mean+standard deviation of two independent experiments for each cell type.

**Figure 8:**
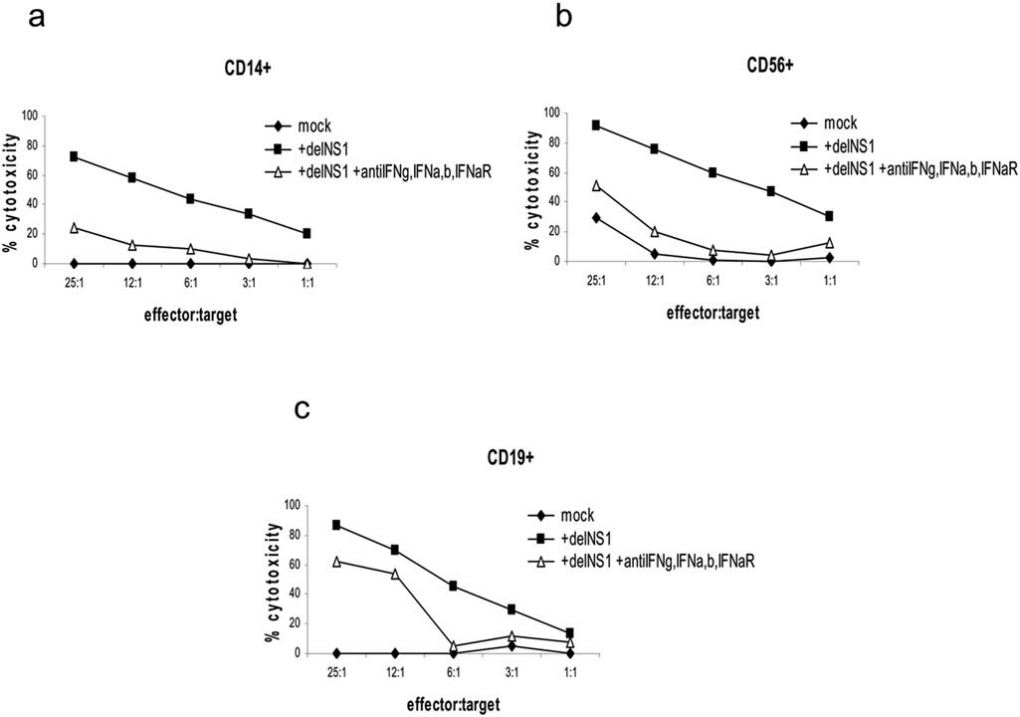
Role of interferon in the anti-tumor cytotoxic immune response by infected monocultures. CD14+ (a), CD56+ (b) and CD19+ cells (c) were infected with delNS1 virus (m.o.i. = 0.02) and cultured for 24 hours with or without anti-IFNα, anti-IFNβ, anti-IFNγ and anti-IFNα-receptor antibodies. Then cells were co-cultured with the target A375 cells for 24 hours and cytotoxicity was assessed. One representative result in percentage of cytotoxicity is given for two experiments which were performed with cells derived from different donors.

In order to determine the contribution of cytokines which mediate the induction of virus-induced killing in PBMCs-subsets we blocked the cytokine production in monocultures. We focused on the IFN system, since IFNs are associated with tumor cell killing. For example, IFNα promotes NK- and CTL-mediated anti-tumor responses [20]. Moreover, type I and type II IFN are associated with adenovirus-mediated oncolysis [21]. The combination of type I and type II IFN blocking antibodies with an IFNα receptor antibody was able to block cytotoxic activity of the CD14+ monoculture by more than 50% (fig. 8). For CD19+ cells the antibody combination was able to lower cytotoxicity by about 30% (range 15–40%), as was the case for CD56+ cells. The CD56+ cells showed a higher variation between different donors (range of 20–80%). Since CD3+ cells neither produced type I nor type II IFN we did not test them in this series of blocking experiments. It should be noted that in order to investigate the effect of IFNα on immune activation rather than on cancer cells we have used the IFN resistant tumor cell line A375. In this cell line direct effects of IFNα are absent [22], which was confirmed in the cell line we used (data not shown).

After the activation phase cytotoxic cells exert the direct cell killing by soluble factors but also by direct binding to the target cell. We blocked mediators associated with cytotoxicity such as NKG2D or TRAIL in isolated subpopulations. In our setting, neither the inhibition of NKG2D in positively isolated CD56+, CD14+ or CD3+CD56- cell populations nor the inhibition of the TRAIL in positively isolated CD14+ cells had any effect on delNS1 induced tumor cell killing. TRAIL inhibition in CD56+ or CD19+ cells showed a slight inhibitory effect of 10% to 15% (data not shown).

### Effect of cytokines in whole PBMC cultures

In monocultures the cocktail of IFN antibodies had an effect in blocking virus induced activation of cytotoxicity. We therefore tested this cocktail in the total culture of PBMCs. However, this antibody combination did not diminish the cytotoxic effect of delNS1 activated PBMCs at any effector to target cell ratio tested (data not shown).

**Figure 9:**
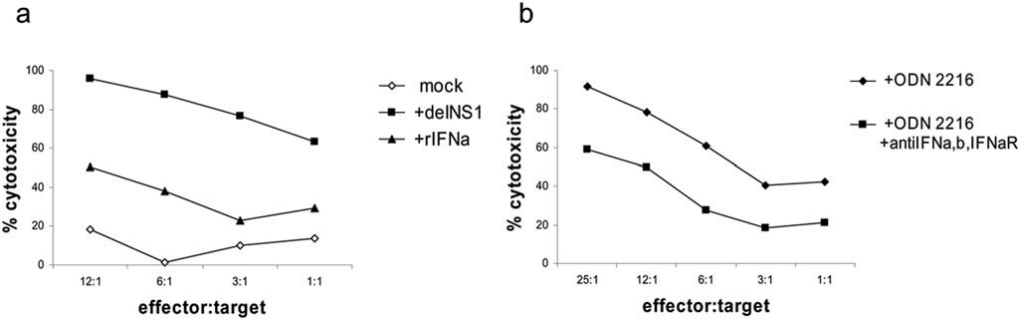
Induction of an anti-tumor cytotoxic immune response by PBMCs infected with delNS1 virus or treated with rIFNα or CpG ODN2216. (a) PBMCs were mock-treated, infected with delNS1 virus (m.o.i. = 0.02) or stimulated with 3000 U/ml rIFNα and incubated for 24 hours. Then PBMCs were co-cultured with the target A375 cells for 24 hours and cytotoxicity was assessed. One representative result in percentage of cytotoxicity is shown of three independent experiments, each performed with PBMCs derived from a different donor. (b) PBMCs were incubated with 12.5 µg/ml CpG ODN2216 with or without addition of anti-IFNα, anti-IFNβ and anti-IFNα-receptor antibodies, and incubated for 24 hours. Then PBMCs were co-cultured with the target A375 cells for 24 hours and cytotoxicity was assessed. One representative result in percentage of cytotoxicity is shown of two independent experiments, each performed with PBMCs derived from a different donor.

Although we were unable to reduce activation of cytotoxicity by blocking IFNs in the total culture of PBMCs, it is likely that IFNs still have an effect in our system, since blockage of IFNs inhibited activation of cytotoxicity in subpopulations. To compare the cytotoxic activation of virus with type I IFN we treated PBMCs either with recombinant IFNα (3,000 U/ml which equals 6,000 pg/ml) or with delNS1 virus at an m.o.i of 0.02. As shown in figure 9a, recombinant IFNα induced a minor increase of cytotoxicity as compared with non treated PBMCs, whereas the tumor killing capacity of delNS1 infected PBMCs reached 98% at the same effector-to-target ratio of 12:1.

Another well known type I IFN inducing agent is CpG, which results in an enhanced cytotoxic activity similar to virus stimulation. We now wanted to test whether the type I IFN system could be circumvented by other mechanisms for activation. We used CpG ODN 2216 known to stimulate NK- and T-cells via IFNα and IFNβ [23]. CpG stimulation induced an efficient cytotoxic activity in PBMCs. In contrast to virus stimulation, CpG stimulation of PBMCs could be partially blocked by the combination of neutralizing antibodies against IFNα/β and the type I IFN receptor from 90% to 60% at the effector-to-target ratio of 25:1 (fig. 9b).

We further tested whether FASL, TRAIL or TNF effector pathways were involved in the cytotoxic activity of virus-activated PBMCs. We treated PBMCs with the respective neutralizing antibodies separately or in combination one hour before co-culture with tumor cells. Neither single application nor combination of anti-FASL, anti-TRAIL and anti-TNF antibodies were able to substantially decrease the cytotoxic effect of virus-activated PBMCs towards tumor cells (data not shown).

## Discussion

Here we demonstrated that stimulation of all predominant cell populations of PBMCs with very low m.o.i of replication deficient delNS1 influenza A virus led to activation of an innate cytotoxic activity of those cells against tumor cells. Virus infection also enhanced the cytotoxic effect of PBMCs derived from cancer patients. Furthermore, the cytotoxic activity was not simply due to allogenicity, since virally enhanced killing was also observed when autologous murine spleen cells and tumor cells were used.

Generally, virus-induced innate cytotoxic immune response is primarily ascribed to NK-cells [24], [25] and to death-related molecules produced by the monocytic lineages [19]. Lately, TRAIL-mediated killing following influenza A virus stimulation was described for a plasmacytoid dendritic cell line [2]. Here we found that influenza A virus induced all major subsets of PBMCs to mediate cytotoxicity. This included CD3+CD56- T-cells and CD19+ B-cells. There are few reports concerning B-cell induced cytotoxicity. One of the few examples is CpG stimulation of B-cells resulting in an autocrine IFN signal and TRAIL expression which mediates cytotoxicity [26]. B-cell mediated cytotoxicity might be supported by the ability of B-cells to express T-cell polarizing cytokines [27] and type I IFN as shown here. The B-cells induced cytotoxicity might now explain the immediate protective effect of these cells during virus infection, which remained unexplained up to now [28]. Obviously, cells with specialized functions in the adaptive immune system appear to have a remnant innate cytotoxic activity upon initial stimulation. This might be relevant in the initial immune response against the pathogen until more differentiated activities of these cells have time to develop.

General reports analyzing virus-induced cytotoxicity have used high multiplicity of infection reflecting the cytotoxic activity of the virus-infected cells [19], [29]. In contrast, we have used a very low multiplicity of infection (as determined on Vero cells). Our work now indicates that a small fraction of infected PBMCs is sufficient for an activation of the majority of uninfected cells, which then promotes the cytotoxic effect. Since the m.o.i. of an in vivo infection is usually low, those secondary immunological phenomena are interesting as they provide insights into the natural host response. In our system virus induced cytotoxicity is dependent on i) the primary activation of cells by the virus, ii) a second wave of immune activation of non-infected cells, and iii) the direct cytotoxic effect of the activated cells.

With respect to primary activation of cells by the virus we found, that live virus is necessary to activate CD3+, CD14+ and CD19+ cells, whereas heat inactivated virus is sufficient to activate CD56+ cells. Heat inactivated virus lost the potential to generate progeny RNA but still binds to the cellular receptor. Thus, the viral haemagglutinin of heat inactivated virus might activate NK cells via binding to the NKp46 receptor as has been shown for haemagglutinin in virus infected cells [30]. Since live virus was necessary in our system for activation of CD19+, CD14+ and CD3+ cell this activation might depend on other mechanisms, such as activation of RIG-1 which is induced by viral RNA [16].

We have further analyzed mediators associated with the second wave of immune activation, the latter which stimulate the cells to exert cytotoxic activation. We demonstrated that different subsets of peripheral blood cells produce distinct cytokine patterns upon virus stimulation indicating differential mechanisms of stimulation. Thus, virus induced activation of cytotoxicity is not restricted to a defined cytokine pattern.

Still, one group of cytokines, which have a relevant role in most of the cellular subsets are IFNs. Type I and type II IFNs are widely known for their involvement in virus induced activation of cytotoxicity [29]. With respect to cancer, IFNs are associated with adenovirus-mediated oncolysis in vivo [21]. Correspondingly, we found that virus induced cytotoxic activation of IFNs producing CD14+, CD19+ or CD56+ cells can be inhibited by antibodies against those IFNs. However, CD3+ cells which do not produce any IFNs were still able to induce cytotoxicity, indicating activation by an IFN unrelated mechanism.

We demonstrated that PBMCs, which contain a mixture of IFN dependent and independent cells, do not ultimately require the presence of IFNs for virus activation of cytotoxicity, since blockage of IFNs did not inhibit virus-induced cytotoxicity. The involvement of the pathways other than IFNα in activation of cytotoxicity in PBMCs was confirmed by experiments, which showed that IFNα alone induced a much weaker cytotoxic response in PBMCs as compared to the virus. In contrast to virus-induced cytotoxicity, cytotoxicity induced by CpG could be blocked by antibodies against type I IFN, indicating a high dependency of this drug on a single pathway. Thus, our finding reveals that delNS1 virus induces a broader range of stimulation than single molecules do. The redundancy of PBMC stimulation by the virus might ensure efficient cytotoxicity of the innate immune response, which might be important to clear viral infection in vivo. Redundant mechanisms for PBMC activation are most likely due to multiple cell-cell interactions of different cell type which can occur in PBMCs. Reciprocal cell-cell interaction which promote virus and non-virus induced activation of cytotoxicity have been demonstrated extensively for NK cells and dendritic cells. In this interaction dendritic cells provide cytokines such as IFNα [20] and IL-12 but also activate NK cells via membrane bound receptors such as NKG2D [29].

We have found that virus-stimulated CD56+ cells are able to produce high amounts of IFNγ. In contrast, Draghi et al. [29] and He et al. [31] demonstrated that CD56+CD3- cells by themselves are unable to generate IFNγ production upon stimulation with influenza virus. However, IFNγ production by NK cells was possible by co-cultivation with dendritic cells producing IL-12 or with CD3+ cells producing IL-2, respectively. None of these cytokines were induced in our CD56+ cell cultures. One difference to the above mentioned studies is the fact that the CD56+ population we isolated still contained CD56+CD3+. Thus, the interaction of CD56+CD3- and CD56+CD3+ cells might lead to IFNγ production via cell-cell interaction. However, CD56+CD3+ are not likely to produce IFNγ on their own, since the CD3+ population containing this subset did not produced IFNγ. An important aspect of our work is the fact that we have used a highly immunogenic, replication defective mutant delNS1 virus, which has been shown to induce a stronger innate immune response than the wild type virus [13]–[15], whereas Draghi et al. [29] and He et al. [31] used an influenza wild type virus. Moreover, we observed a cytokine response when virus was used at low m.o.i. of 0.02, whereas other authors only observed a cytokine response at an m.o.i. of 10 or higher [29]. It is known that cytokine patterns of immune cells following influenza A virus stimulation are critically dependent on the m.o.i. [32]. At a high m.o.i. all cytokines expressing cells might be directly stimulated and possibly lysed by the virus. Being able to use the virus at a low m.o.i. we allowed a second wave of cytokine stimulation. This secondary cytokine wave is produced by cells not infected by virus, but activated by cytokines and cell-cell interactions, which have been induced previously by the initial infection. The cytokine composition of this second wave seems to be different from that of the first wave.

With respect to the mechanism of the virus induced cytotoxic effect our transwell analysis indicates that cell-cell interaction mediate around half of the effect, whereas the other half was mediated by soluble factors in the supernatant. Although we assayed for a number of known cytotoxicity- associated soluble factors such as TNF, TRAIL, FASL, NKG2D, perforin or granzyme B, we could not identify single factors responsible for this effect. It should be noted that we have used a type I IFN resistant target cell line, which restricts the latter pathways. In contrast, TRAIL in IFN sensitive target cells lines has been found to play a role in virus stimulated cells, as has been shown for Newcastle Disease virus (NDV) induced tumoricidal effects at high m.o.i. [19]. Our analysis indicates that other factors are able to compensate whenever TRAIL is not functional.

What are the implications of these findings for viral immunology? First, during regular influenza A virus infection all activated immune cells might initially exert cytotoxic functions thus limiting initial viral spread by killing of virus infected cells. Second, we regard the influenza A virus (delNS1) suitable for activation of a cytotoxic immune response to cancer. This might be achieved by ex vivo stimulation of peripheral blood cells with the virus and subsequent adoptive transfer of viral activated cells into the patient. Alternatively, delNS1 virus might be injected as oncolytic viro-immunotherapy directly into the tumor with the intention to induce cytotoxic activity in the anergic immune cells present in the tumor tissue. In conclusion our data highlight the pleiotropic functions of immune cells and provide further evidence of a close inter-relation of the adaptive and the innate immune system.

## Materials and Methods

### Ethics Statement

The use of human peripheral blood has been approved by the ethical committee of the Medical University of Vienna, Austria. The use of animals has been approved by the Ministry of Science of Austria.

### Cells and Viruses

Sk-Mel 28 and A375 (melanoma), CaCo-2 (colon cancer), MCF-7 (breast carcinoma) and C-26 (murine colon cancer) were obtained from the American Type Culture Collection (ATCC, Rockville, MD). Cells were grown in DMEM (Bio Whittaker, Belgium) supplemented with 2 mM L-glutamine, 100 U/ml penicillin, 100 µg/ml streptomycin and 10% heat-inactivated FCS (Gibco BRL/Invitrogen™, California). GSJO, a primary medullary thyroid carcinoma cell line (A.S, Department of Surgery, Medical University Vienna) was cultured in RPMI-1640 medium supplemented with glutamax-I at 50 µg/ml (Gibco BRL, Life Technologies, Scotland), gentamicin (Gibco BRL) and 10% heat-inactivated FCS. Vero cells (EC ACC, 88020401) adapted to grow in serum-free medium were maintained in serum-free OPTIPRO medium (Invitrogen). The cells were maintained in a humidified 5% CO_2_ atmosphere at 37°C. Cell stocks were screened for mycoplasma by the polymerase chain reaction method (Boehringer Mannheim, Germany).

Influenza A/PR/8 (PR8) virus and delNS1 virus were generated as described [33]. Heat-inactivation of PR8 virus was performed at 56°C for 30 min. For propagation of the viruses, Vero cells were infected at a multiplicity of infection (m.o.i.) of 0.1 and incubated in OPTIPRO medium containing 5 µg/ml trypsin (Sigma Aldrich) at 37°C for 2–3 days. Virus concentrations were determined by plaque assay on Vero cells.

### Infection of PBMCs and monocultures with influenza virus

After written informed consent, peripheral blood mononuclear cells (PBMCs) from healthy donors and cancer patients were isolated using Ficoll-Hypaque (Amersham Pharmacia Biotech AB, Sweden) density gradient centrifugation. To deplete CD56+, CD3+, CD14+ and CD19+ cells PBMCs were separated with the corresponding microbeads (Miltenyi Biotec, Germany). All depletions were carried out according to the manufacturer's instructions. Cell depletions were confirmed by flow cytometry.

Monocultures of human CD56+, CD3+, CD14+ and CD19+ cells were obtained by magnetic separation using cell specific isolation kits (Milteny Biotec, Germany) following the manufacturer's instructions. As determined by flow cytometry, the procedure resulted in >90% pure cell isolates. Purity of CD56+ cells was 93.6+/−3.7% and contained 15.2 +/−9.6% CD56+/CD3+ cells, 1.5+/−0.8% CD14+ cells and 0.34+/−0.47% CD19+cells. Purity of CD19 cells was 93+/−2.5% and contained than 0.5+/−0.4% CD56 cells, 3.7+/−1.8% CD3+ cells 1.1+/−1.0% CD14+ cells. Purity of CD14+ cells was 93.3+/−3.1% and contained 0.3+/−0.3% CD19+ cells, 1.8+/−1.0% CD3+ and 1.16+/−1.1% CD 56+ cells. Purity of CD3+ cells was 96.4+/− 1.5% and contained 4.7 +/−2.6% CD56+/3+, 0.5% CD14+ cells and un-detectable amounts of CD19+ cells. CD3+CD56- cells were obtained by positive selection for CD3 cells followed by depletion with CD56 specific antibody. Purity of CD3+CD56- cells was 92+/−1% and contained 1.8 +/−0.9% CD14+ cells, 2.1+/−0.5% CD56+ cells and 0.5+/−0.3% of CD19+ cells.

Immediately after isolation PBMCs were infected with delNS1 or PR8 virus at an m.o.i. of 0.02 in serum-free OPTIPRO medium containing 4 mM glutamine. After incubation for 30 min. at room temperature, the inoculums were removed and cells were washed. Uninfected control cells were treated in the same manner except that no virus was added (mock infection). Virus-activated and non-activated PBMCs were cultured at a density of 5×10^6^ cells/ml in RPMI-1640 medium supplemented with 2 mM glutamine and 10% heat inactivated FCS for 24 h prior to co-culture with tumor cells.

To determine the amount of the infectious viral particles in the supernatant of the virus-activated PBMCs, 1ml of supernatant was centrifuged at 25 000 r.p.m. for 18h, at +4°C. The pellet was resuspended in 500 µl of serum-free OPTIPRO medium containing 4 mM glutamine and 100 µl of that suspension was used to determine the viral concentration via plaque assay on Vero cells.

For the stimulation of PBMCs with the CpG reagent ODN2216, cells were cultured for 24 h (4×10^6^/ml) in the presence of CpG ODN 2216 (12.5 µg/ml; VBC-Biotech, Austria) in RPMI-1640 medium supplemented with 2 mM glutamine and 10% heat inactivated FCS.

To activate PBMCs with INFα, the recombinant form (Roche, Switzerland) was added at the final concentration of 3000 IU/ml 16 h before cells were co-cultured with tumor cells.

### Animals

Six week old BALB/c female mice were purchased from Himberg (Austria). All animals were maintained at standard conditions and fed a standard diet and water *ad libitum*.

Spleens were removed from mice under aseptic conditions and homogenized. After the lysis of erythrocytes, cells were washed and resuspended in serum-free OPTIPRO medium and infected with virus as described above.

### Cytotoxicity assay and inhibition experiments

Tumor cells were seeded in 96-well flat-bottom plates at a density of 5×10^3^/well and left for several hours to adhere. The cytotoxicity of influenza virus activated or non-activated PBMCs was tested in duplicates against tumor cells at different effector-to-target cell ratios after 24 h of co-culture. The tumor cell viability was determined by colorimetric Easy for You Assay Kit (EZ4U Kit, Biomedica, Austria) according to the manufactureŕs instructions and absorbance at 450 nm was measured spectrophotometrically using a Dynatech Microplate Reader 5000 (Dynatech Laboratories Inc., Chantilly, VA). Results were calculated as the percentage of viability equaling the (OD of co-culture–OD of PBMCs)/OD tumor cells×100.

To determine the sensitivity of cancer cells to the infection with delNS1 virus, the tumor cells A375, Sk-Mel 28, CaCo-2 and MCF-7 were seeded as described above and infected with a m.o.i. of 0.2 and 0.02. The infected cells were cultivated in RPMI-1640 medium supplemented with 2 mM glutamine and 10% heat inactivated FCS. The viability of the cells was determined 24h after infection by colorimetric Easy for You Assay Kit.

For inhibition experiments, azide-free anti-TRAIL antibody 2E5 (5 µg/ml; Alexis, San Diego, CA) was added to effector cells 45 min before addition of target cells. Neutralization of NKG2D was comparably achieved by 20 µg/ml of anti-NKG2D mAb (R&D). Efficient blocking using those antibodies against TRAIL and NKG2D has been proven in by Stary et al. [5]. FAS ligand mediated killing was inhibited by anti-FAS-L mAb NOK-1 (20 µg/ml; Santa Cruz Biotechnology, Santa Cruz, CA). TNF activity was blocked by adding human TNF RII/TNFRSF1B/Fc chimera (20 µg/ml; R&D) to effector cells 1 h before co-culture with target cells. For blocking of IFNα/β function, a combination of polyclonal rabbit anti-IFNα (5,000 neutralizing U/ml) and rabbit anti-IFNβ (2,000 neutralizing U/ml) antibodies together with 20 µg/ml of a mouse anti-human IFNα/β receptor chain 2 mAb were used (all from PBL, New Brunswick, NJ). Efficient blocking using those antibodies against type I IFN has been proven by Rothenfusser et al. [23]. For blocking of IFNγ mouse anti-human IFNγ Ab (R&D) was applied. IFNα/β and IFNγ function was blocked immediately after infection of PBMCs with delNS1 virus.

Neutralisation of delNS1 virus was performed using sheep anti-serum reagent to hemagglutinin (HA) of A/PR/8/34 virus (03/242, NIBSC, Hertfordshire, UK) used at a final dilution of 1:40. The blocking efficacy of the HA anti-serum was proven by the inhibition of the Vero cells killing mediated by the infection with delNS1 at the m.o.i. of 0.2. (fig. S3). For the neutralisation of possible viral particles present on virus-activated PBMCs, PBMCs were activated with the delNS1 as described above and HA anti-serum was added to the PBMCs culture 1h prior the co-cultivation with the tumor cells. The cytotoxicity of influenza virus activated PBMCs was tested in duplicates against tumor cells at the effector:target ratio = 25:1 after 24h of co-culture by means of colorimetric Easy for You Assay Kit.

### Cell characterization by flow cytometry

The phenotype of delNS1 activated versus non-activated PBMCs was determined by fluorescence analysis. Cells (3×10^5^) were resuspended in 50 µl of assay buffer (PBS, 2% FCS and 1% sodium azide) and incubated for 30 min at 4°C with fluorescein isothiocyanate (FITC) or phycoerythrin (PE) labeled monoclonal antibodies specific to cell surface markers. Monoclonal antibodies specific for, CD3, CD14, CD56, CD19, perforin (Beckman Coulter, France), granzyme B (Serotec, England) and NKG2D (BD Pharmingen, Belgium) and the indicated isotype controls were used to characterize cells. Cellular fluorescence was analyzed by EPICS XL-MCL flow cytometer (Coulter, USA) with EXPO32 software (Beckman Coulter, France). 10,000 events were acquired for each sample and the percentage of positive cells was reported.

### Cytokine detection after infection with delNS1

PBMCs and monocultures of CD3+, CD14+, CD56+ or CD19+ cells isolated from healthy donors were infected with delNS1 (m.o.i. = 0.02) and cultured for 24 hours in RPMI 1640 medium containing 10% FCS. The supernatant was then screened with Luminex® for IL-1β, IL-4, IL-6, IL-8, IL-10, IL-12(p70), IFNγ and TNFα (Upstate, Temecula, CA). IFNα was detected by ELISA (PBL Interferon Source, NJ).

### Transwell cultures

Transwell cultures were established in 12 well plates (Costar®, Corning Inc., USA) with Falcon™ inserts (1 µm pore size, Becton Dickinson, France). To keep the effector:target ratio = 25:1 constant the number of PBMCs placed into the top chamber and the number of tumor cells in the bottom chamber was adjusted to account for the larger volumes used. The total volume was 2 ml of medium. Controls consisting of cultures without transwells were included. To retrieve supernatant only, the PBMCs with or without delNS1 infection were cultured as described above. Supernatant was transferred directly to tumor cell cultures. All cultures were incubated for 24 h in a total volume of 2 ml.

## Supporting Information

Figure S1 Influenza A delNS1 does not induce tumor lysis. A375, CaCo-2, Sk-Mel 28 MCF-7 were infected with the delNS1 virus at the m.o.i. of 0.2 and 0.02 or not infected (mock). The viability of the cells was determined 24h after infection by means of colorimetric Easy for You Assay Kit.
(0.08 MB TIF)

Figure S2 Neutralizing influenza heamagglutinin (HA) anti-serum does not reduce cytotoxicity of delNS1-virus stimulated PBMCs. PBMCs were mock-treated or infected with delNS1 virus (m.o.i. = 0.02) and were incubated for 24 hours. HA anti-serum was added to the PBMCs culture 1h prior the co-cultivation with the tumor cells. The viability of the tumor cells was determined after 24h of co-culture. One representative result out of three independent experiments, each performed with PBMCs derived from a different donor, is shown.
(0.71 MB TIF)

Figure S3 Neutralizing influenza heamagglutinin (HA) anti-serum inhibits Vero cells killing mediated by the infection with delNS1. The neutralizing HA-anti serum was added to the virus suspension 1h prior infection of the Vero cells. The Vero cells were infected at the m.o.i. of 0.2 either with the untreated or with the HA-anti serum neutralized delNS1 virus. The control group was left uninfected (mock). The viability of the Vero cells was determined 48h after infection by means of colorimetic Easy for You Assaz Kit.
(0.05 MB TIF)
