# Supplementary figures and images for: Influenza A Virus Induces an Immediate Cytotoxic Activity in All Major Subsets of Peripheral Blood Mononuclear Cells
Source: PLoS One. 2009 Jan 6;4(1):e4122. doi: 10.1371/journal.pone.0004122 (PMC2610492; doi:10.1371/journal.pone.0004122)

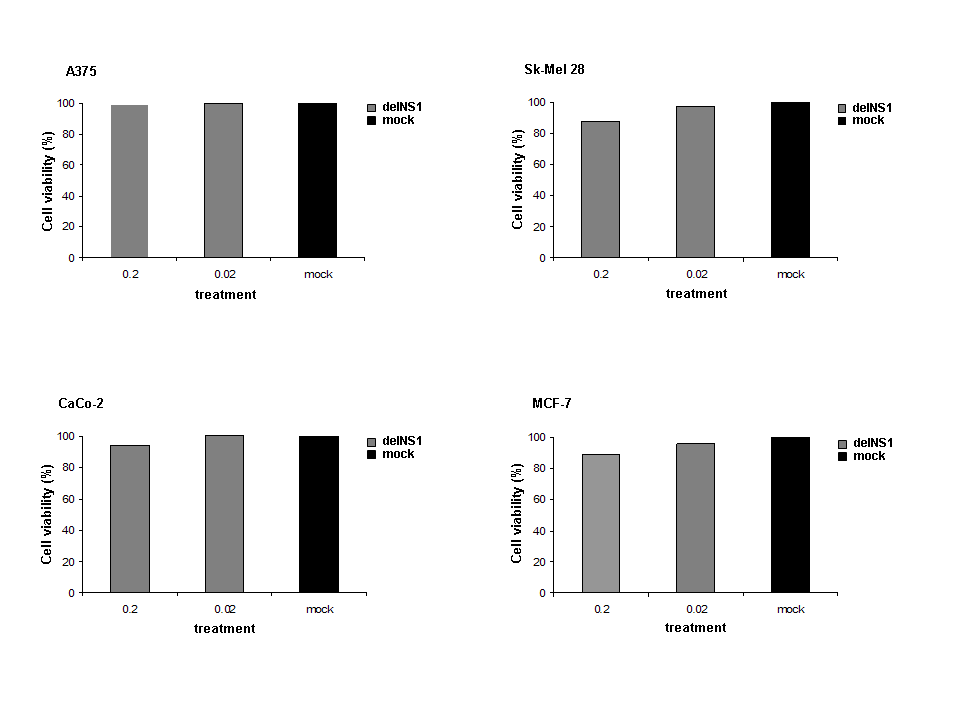

Supplement: Figure S1 — Influenza A delNS1 does not induce tumor lysis. A375, CaCo-2, Sk-Mel 28 MCF-7 were infected with the delNS1 virus at the m.o.i. of 0.2 and 0.02 or not infected (mock). The viability of the cells was determined 24h after infection by means of colorimetric Easy for You Assay Kit. (0.08 MB TIF) [file pone.0004122.s001.tif]

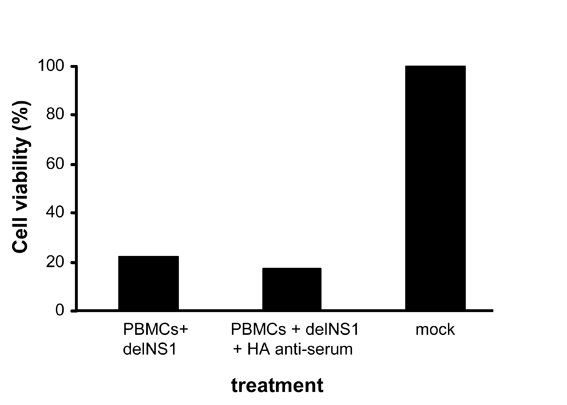

Supplement: Figure S2 — Neutralizing influenza heamagglutinin (HA) anti-serum does not reduce cytotoxicity of delNS1-virus stimulated PBMCs. PBMCs were mock-treated or infected with delNS1 virus (m.o.i. = 0.02) and were incubated for 24 hours. HA anti-serum was added to the PBMCs culture 1h prior the co-cultivation with the tumor cells. The viability of the tumor cells was determined after 24h of co-culture. One representative result out of three independent experiments, each performed with PBMCs derived from a different donor, is shown. (0.71 MB TIF) [file pone.0004122.s002.tif]

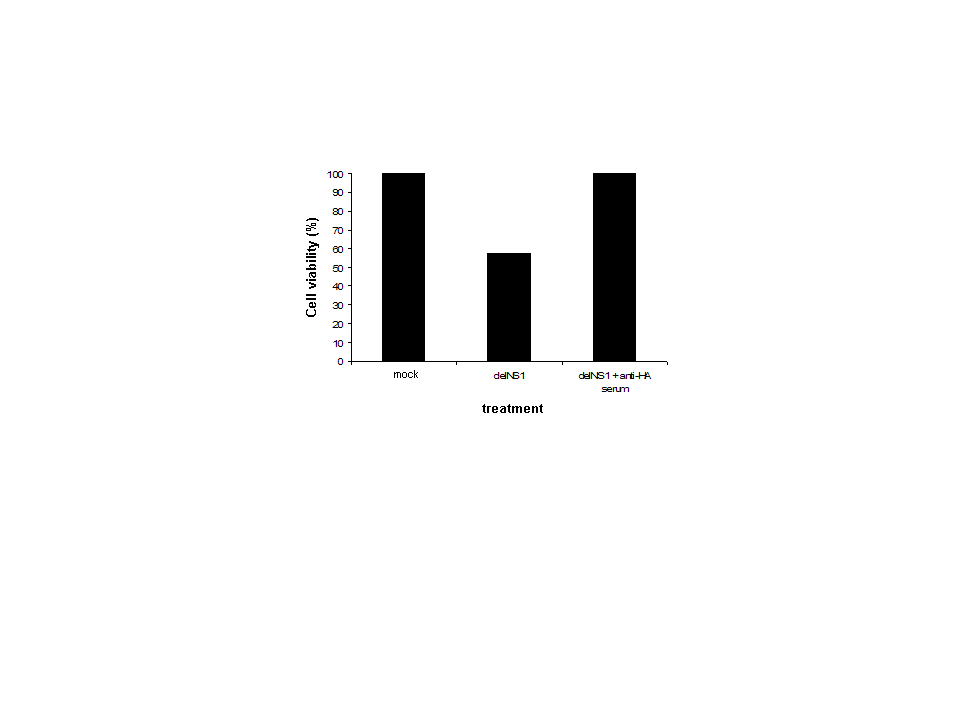

Supplement: Figure S3 — Neutralizing influenza heamagglutinin (HA) anti-serum inhibits Vero cells killing mediated by the infection with delNS1. The neutralizing HA-anti serum was added to the virus suspension 1h prior infection of the Vero cells. The Vero cells were infected at the m.o.i. of 0.2 either with the untreated or with the HA-anti serum neutralized delNS1 virus. The control group was left uninfected (mock). The viability of the Vero cells was determined 48h after infection by means of colorimetic Easy for You Assaz Kit. (0.05 MB TIF) [file pone.0004122.s003.tif]
